# TIGIT acts as an immune checkpoint upon inhibition of PD1 signaling in autoimmune diabetes

**DOI:** 10.3389/fimmu.2024.1370907

**Published:** 2024-03-12

**Authors:** Prerak Trivedi, Gaurang Jhala, David J. De George, Chris Chiu, Claudia Selck, Tingting Ge, Tara Catterall, Lorraine Elkerbout, Louis Boon, Nicole Joller, Thomas W. Kay, Helen E. Thomas, Balasubramanian Krishnamurthy

**Affiliations:** ^1^ Immunology and Diabetes Unit, St Vincent’s Institute, Fitzroy, VIC, Australia; ^2^ Department of Medicine, St Vincent’s Hospital, The University of Melbourne, Fitzroy, VIC, Australia; ^3^ JJP Biologics, Warsaw, Poland; ^4^ Department of Quantitative Biomedicine, University of Zurich, Zurich, Switzerland

**Keywords:** type 1 diabetes, TIGIT, CD8+ T cell, immune checkpoint, NOD mouse

## Abstract

**Introduction:**

Chronic activation of self-reactive T cells with beta cell antigens results in the upregulation of immune checkpoint molecules that keep self-reactive T cells under control and delay beta cell destruction in autoimmune diabetes. Inhibiting PD1/PD-L1 signaling results in autoimmune diabetes in mice and humans with pre-existing autoimmunity against beta cells. However, it is not known if other immune checkpoint molecules, such as TIGIT, can also negatively regulate self-reactive T cells. TIGIT negatively regulates the CD226 costimulatory pathway, T-cell receptor (TCR) signaling, and hence T-cell function.

**Methods:**

The phenotype and function of TIGIT expressing islet infiltrating T cells was studied in non-obese diabetic (NOD) mice using flow cytometry and single cell RNA sequencing. To determine if TIGIT restrains self-reactive T cells, we used a TIGIT blocking antibody alone or in combination with anti-PDL1 antibody.

**Results:**

We show that TIGIT is highly expressed on activated islet infiltrating T cells in NOD mice. We identified a subset of stem-like memory CD8+ T cells expressing multiple immune checkpoints including TIGIT, PD1 and the transcription factor EOMES, which is linked to dysfunctional CD8+ T cells. A known ligand for TIGIT, CD155 was expressed on beta cells and islet infiltrating dendritic cells. However, despite TIGIT and its ligand being expressed, islet infiltrating PD1+TIGIT+CD8+ T cells were functional. Inhibiting TIGIT in NOD mice did not result in exacerbated autoimmune diabetes while inhibiting PD1-PDL1 resulted in rapid autoimmune diabetes, indicating that TIGIT does not restrain islet infiltrating T cells in autoimmune diabetes to the same degree as PD1. Partial inhibition of PD1-PDL1 in combination with TIGIT inhibition resulted in rapid diabetes in NOD mice.

**Discussion:**

These results suggest that TIGIT and PD1 act in synergy as immune checkpoints when PD1 signaling is partially impaired. Beta cell specific stem-like memory T cells retain their functionality despite expressing multiple immune checkpoints and TIGIT is below PD1 in the hierarchy of immune checkpoints in autoimmune diabetes.

## Introduction

Insulin-producing beta cells are destroyed by self-reactive CD8+ T cells in autoimmune diabetes. From the initiation of autoimmunity to clinical diagnosis of autoimmune diabetes takes years in humans and months in mice. It is postulated that immune regulatory mechanisms keep beta-cell-specific T cells in check, and this is one of the main reasons why there is a long lag time for beta-cell destruction in autoimmune diabetes. Understanding and harnessing these immunoregulatory mechanisms can help in developing future immunotherapies for type 1 diabetes.

One of the key immunoregulatory mechanisms by which CD8+ T cells are controlled is via immune checkpoint or inhibitory receptor engagement (1, 2). Upon chronic antigen stimulation, T cells upregulate inhibitory surface receptors including CTLA4, PD1, LAG3, TIGIT, and TIM3 (3, 4). These inhibitory receptors are comprised of a ligand binding extracellular domain and an intracellular signaling domain that recruits effector proteins to prevent T-cell activation (2). Tumors exploit immune checkpoint pathways to escape from the immune system by preventing the activation of tumor-antigen-recognizing T cells. Blockade of immune checkpoints like PD1 and CTLA4 results in reinvigoration of the T-cell response against tumors, and checkpoint inhibitor antibodies are now widely used in cancer immunotherapy (5, 6).

Chronic antigen stimulation in the islets leads to the differentiation of CD8+ T cells into two major subsets resembling those found in tumors and chronic viral infections (7–9). These include progenitor/stem-like memory T cells and terminally differentiated/exhausted T cells (10, 11). Stem-like memory T cells are marked by the expression of the inhibitory receptor PD1 and a key transcription factor, TCF1, which confers T cells with the ability for self-renewal (12). Terminally exhausted T cells are marked by the expression of TIM3 in addition to multiple inhibitory receptors including PD1 and TIGIT, but they lose TCF1 expression (12). Stem-like memory T cells have long-term survival, retain their functionality, and can give rise to effector T cells. In tumors and chronic viral infections, blockade of PD1 induces proliferation and differentiation of these stem-like memory T cells, giving rise to the effector population that controls tumors and viral infections (11, 13, 14). In parallel, inhibition of PD1 results in rapid diabetes in NOD mice (15–17) and humans (18) with pre-existing autoimmunity against beta cells. Inhibiting LAG3 in CD8+ T cells also results in accelerated diabetes in NOD mice (19), indicating that both PD1 and LAG3 restrain T cells in the islets.

TIGIT is also expressed by islet-infiltrating T cells (8, 9, 19). TIGIT binds with high affinity to CD155 (20) and with low affinity to CD112 (21). CD226, a co-stimulatory molecule expressed on T cells, competes with TIGIT for ligand binding. TIGIT can inhibit T-cell activation by competing with CD226 for its ligand and by negatively regulating T-cell receptor signaling through its intracellular signaling domain (22–24). CD226-deficient NOD mice have reduced incidence of diabetes (25), and polymorphisms in the *CD226* gene are associated with type 1 diabetes in humans (26). This evidence suggests a role for the TIGIT-CD226 signaling axis in autoimmune diabetes. Despite this evidence, it remains unknown if TIGIT acts as an immune checkpoint in autoimmune diabetes. We studied the expression and function of TIGIT-CD226 in autoreactive CD8+ T cells in the NOD mouse model of type 1 diabetes to better understand the role of TIGIT and the hierarchy of immune checkpoints in autoimmune diabetes.

## Materials and methods

### Mice

NOD/Lt mice were bred and maintained at Bioresources Centre, St, Vincent’s Hospital. All animal studies were approved by the institutional animal ethics committee. Female mice were used for all experiments.

### Islet isolation and preparation of single-cell suspensions

Mouse islets were isolated using collagenase P (Roche) and Histopaque-1077 density gradient (Sigma-Aldrich) as described previously (27). Isolated islets were dispersed into single-cell suspension using bovine trypsin (Calbiochem) and 2 mM EDTA in PBS. Single cells were washed and then incubated in complete RPMI for 1 h at 37°C before being processed for further treatments or staining.

Single-cell suspension from spleens was prepared by mechanical disruption of tissue and filtering through 70-μm cell strainers. Red blood cell lysis was performed using Tris-buffered ammonium chloride for 3–4 min. Cells were then resuspended in 0.5% FCS and 4 mM EDTA in PBS (MACS buffer) before processing for tetramer staining and magnetic bead enrichment.

### Cell sorting, library preparation, and sequencing for sc-RNAseq

For sc-RNAseq analysis, live CD45+ cells were FACS sorted from dispersed islets pooled from two to three NOD mice (15–16 weeks of age) per sample. Sorted CD45+ cells were washed and resuspended in RPMI cell culture medium (Gibco) containing 10% FCS at a density of 1,200 cells/μl and loaded on to Chromium Controller (10x Genomics) (9). Three samples from two independent experiments were processed further using Chromium Single cell 3’ Gel bead kit (v 3.1) and library construction kit as per manufacturer’s instructions. The libraries were quantified using the Agilent Bioanalyzer High-Sensitivity Chip and sequenced on the Illumina NovaSeq PE150 platform (Novogene AIT Genomics, Singapore). The single-cell expression data used were from GSE247956.

### Single-cell RNA sequencing and analysis

The scRNA-seq reads were aligned to the mm10 reference genome and quantified using cellranger count (10x Genomics, version cellranger-6.0.1). We obtained at least a median of 1,572 genes per cell and mean of 29,750 unique transcripts per cell. All analyses were performed in RStudio (version 2023.06.2 + 561 “Mountain Hydrangea” Release) and Seurat (version 4.3.0.1) unless specified. Cell-specific filtering of datasets for each of the samples was performed by retaining cells with RNA features between 200 and 5,000, RNA count <6,000, <5% mitochondrial RNA, and >5% ribosomal RNA. Gene-specific filtering was performed by removing Malat1, mitochondrial, and ribosomal genes. Doublets were removed by using DoubletFinder with parameters pN = 0.25, pK = 0.29, and 7.6% expected multiplet rates, and using the first 30 PC. Individual datasets were normalized by using the default parameters of the SCTransform function. Integration of datasets was performed by using functions SelectIntegrationFeatures, PrepSCTIntegration, FindIntegrationAnchors, and IntegratedData.

The minimum cumulative number of PCs that covered >90% variance was calculated by using the ElbowPlot function, which was used for the included dimension in functions RunUMAP and FindNeighbours. The resolution for function FindCluster was guided by obtaining biologically meaningful clusters from visualizing the expression of marker genes with FeaturePlot and assessing the differentially expressed genes by the output of the Wilcoxon rank sum test in FindAllMarkers. Re-clustering of subset with clusters of interest was performed iteratively from subsetting T cells, CD8 single positive, NOD genotype, CD44hi, and PD1hi by running functions RunPCA, RunUMAP, FindNeighbours, FindClusters, and FindMarkers as discussed above. The final clustering was a result of using first 15 PC with clustering resolution at 0.8. Ggplot2 package (version 3.4.3) was used for visualization.

### Tetramer staining and magnetic-bead-based enrichment

Magnetic-bead-based tetramer enrichment assay has been described previously (28). Briefly, single-cell suspensions were stained with phycoerythrin (PE) labeled IGRP_206–214_ (VYLKTNVFL) H2-Kd tetramer (ImmunoID, Parkville, Australia) for 1 h at 4°C, then washed and stained with anti-PE magnetic beads (Miltenyi Biotech) for 20 min at 4°C. Samples were then washed and run on the AutoMACSpro (Miltenyi Biotech) separator to enrich IGRP tetramer-specific T cells. These enriched T cells were then stained for cell surface markers to perform flow cytometry analysis.

### Flow cytometry

Spleen or islet single-cell suspensions were stained for 30 min at 4°C with (**Supplementary Table S1**) anti-CD11b (1:200; eFluor450; eBioscience, 48-0012-82), anti-CD11c (1:200; eFluor450; eBioscience, 48-0114-82), anti-Ly6g/Ly6c (1:200; eFluor450; eBioscience, 48-5931-82), anti-CD45R/B220 (1:200; eFluor450; eBioscience, 48-0452-82), anti-CD3 (1:100; V500; BD, 560771), anti-CD4 (1:400; APC-Cy7; BD, 552051), anti-CD8 (1:300; BV711; Biolegend, 100759), anti-CD44 (1:400; PE-Cy7; Biolegend, 103030), anti-PD1 (1:300; BV605; Biolegend, 135220), anti-Slamf6 (1:100; FITC; Miltenyi, 130-118-597), anti-TIGIT (1:300; PE Dazzle 594; Biolegend, 142110), anti-CD226 (1:300; BV785; Biolegend, 133611), and anti-CD155 (1:200; BV605; Biolegend, 131519). B220, CD11c, CD11b, and Ly6G were used for gating out non-T cells, and CD3 was used to identify T cells in all experiments (**Supplementary Figure S1A**). Naive CD8+ T cells from islets were used for gating controls for SLAMF6, TIGIT, and CD226 staining (**Supplementary Figures S1B, C**). For B cells and dendritic cells, the CD45+ cells were gated, and CD11b- and CD11c-positive cells were identified as dendritic cells, and B220 expressing CD11c- and CD11b-negative cells were identified as B cells (**Supplementary Figure S2A**). For islet cells, the CD45− cells were gated and beta cells identified by their high autofluorescence (**Supplementary Figure S2B**).

For all intracellular staining experiments, after cell surface staining, the Foxp3 transcription factor buffer staining kit (eBioscience, 00-5523-00) was used to fix and permeabilize the cells. For intracellular staining, anti-EOMES (1:300; PE-Cy7; eBioscience, 25-4875-82), anti-Ki-67 (1:400; PE-Cy7; eBioscience, 25-5698-82), or anti-IFNγ (1:200; FITC; Invitrogen, 11-7311-82) were used. All flow cytometry data were acquired on Cytek Aurora (spectral analyzer) and analyzed using Flowjo V10 software.

### Cell stimulation and intracellular cytokine staining

Single cells were resuspended in complete RPMI medium in 96-well plates and stimulated with PMA (50 ng/ml) and ionomycin (1,000 ng/ml) for 4 h at 37°C in the presence of GolgiPlug™ (BD Biosciences). All cell surface markers were stained first; then, cells were fixed and permeabilized with the Foxp3/transcription factor staining kit (eBiosciences) and stained with anti-IFNγ.

### *In vivo* treatment of mice

For diabetes induction by immune checkpoint blockade, anti-PDL1 (M1H5) (250 ug/mouse) three times (17) or anti-TIGIT (1B4) (29) (100 ug/mouse) four times was injected intraperitoneally in 15–18-week-old female NOD mice.

For the combination, one dose of anti-PDL1 (250 ug/mouse) was administered, and then, from the next day onwards, three doses of anti-TIGIT (100 ug/mouse) were given to 11–13-week-old female NOD mice.

To investigate the proliferation of islet-infiltrating T cells, two doses of anti-PDL1 (250 ug/mouse) or anti-TIGIT (100 ug/mouse) were given to 15–18-week-old NOD female mice.

### Diabetes monitoring

Mice were monitored for diabetes by measuring blood glucose level using Advantage II glucose strips (Roche). Two consecutive readings of 15 mM were considered diabetic.

### Statistical analysis

All statistical analyses were performed using GraphPad Prism 8 software (GraphPad, USA). All data shown as a bar graph are presented as the mean ± SD. A p-value of <0.05 was considered to be significant. Student’s t-test was used for comparisons between two groups. Multiple comparisons were performed using one-way ANOVA with Tukey or Bonferroni *post-hoc* test. Diabetes incidence was compared using log-rank (Mantel–Cox) test.

## Results

### TIGIT is expressed on islet-infiltrating T-cell subsets

**Figure 1:**
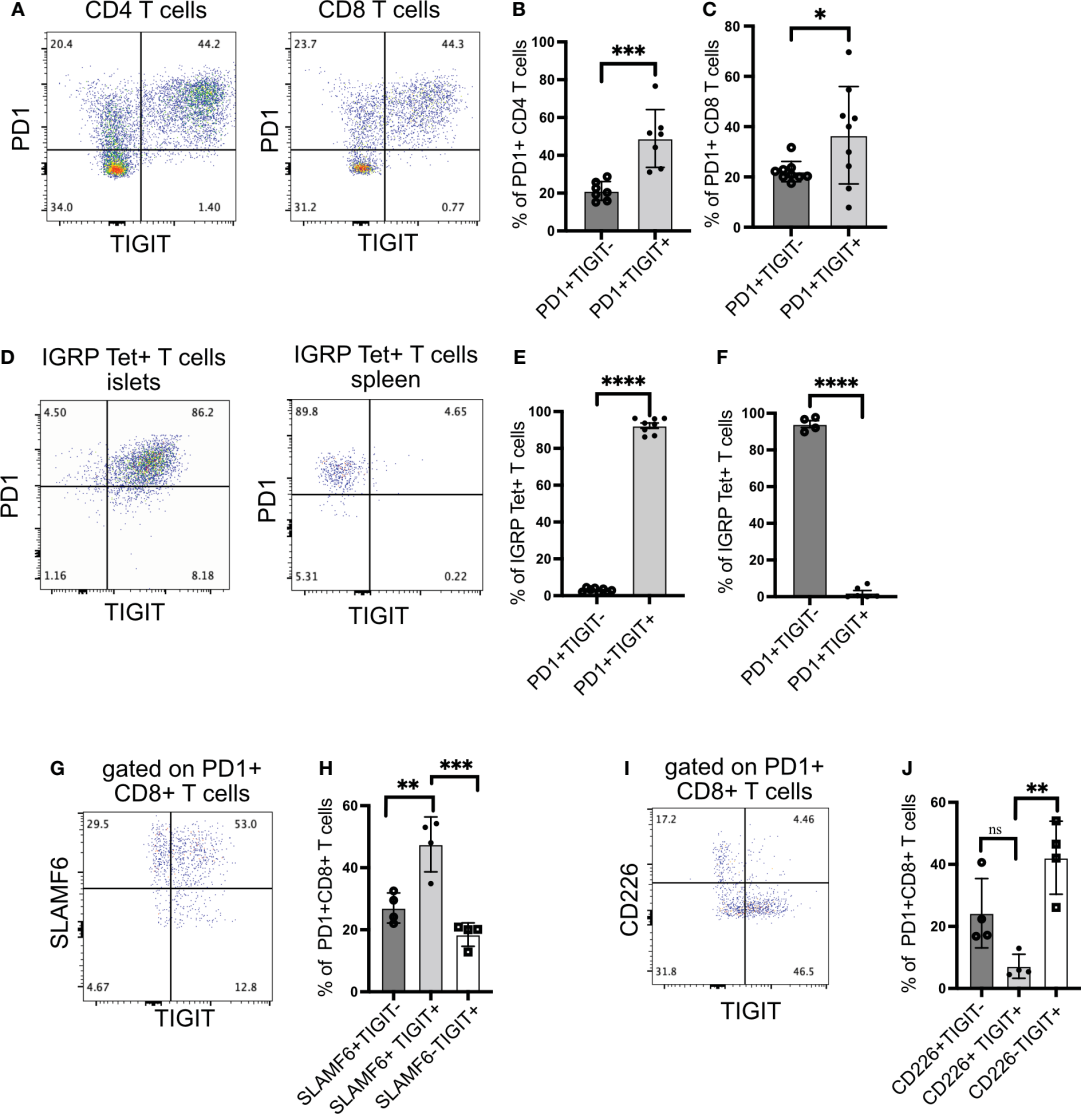
TIGIT and CD226 expression on islet-infiltrating T cells. **(A–C)** The frequency of PD1 and TIGIT expressing islet-infiltrating T cells (n= 7–9 mice/group). Data show CD4+ T cells **(B)** and CD8+ T cells **(C)** in islet CD45+ cells. Representative data **(A)** and pooled data showing mean ± SD and individual mice from two to three independent experiments **(B, C)**. ***p<0.001 and *p<0.05 unpaired Student’s t-test. **(D–F)** The frequency of IGRP_206-214_-specific CD8+ T cells expressing PD1 and TIGIT in islets **(D, E)** and spleen **(D, F)**. Representative data **(D)** and pooled data show mean ± SD and individual mice from two independent experiments (n=8 mice/group) for islets and (n=4–6 mice/group) for spleen. ****p<0.0001 unpaired Student’s t-test. **(G, H)** Frequency of SLAMF6 and TIGIT-expressing PD1+CD8+ T cells from islets (n=4 mice/group). Representative data **(G)** and pooled data **(H)** showing mean ± SD of the frequency of cells in each quadrant in panel **(G)**, with individual mice shown. **p<0.01 and ***p<0.001 one-way ANOVA Tukey’s multiple comparison test. **(I, J)** Frequency of CD226 and TIGIT-expressing PD1+CD8+ T cells from islets (n=4 mice/group). Representative data **(I)** and pooled data showing mean ± SD and individual mice **(J)**. **p<0.01, ns=not significant one-way ANOVA Tukey’s multiple comparison test. All mice in **Figure 1** were 16–20-week-old female NOD mice.

To understand the role of TIGIT as an immune checkpoint in autoimmune diabetes, we first investigated the expression of TIGIT in islet-infiltrating T cells of NOD mice. TIGIT was expressed on both CD4+ T cells (**Figures 1A, B**) and CD8+ T cells (**Figures 1A, C**). A higher proportion of PD1+ T cells in the islets were TIGIT+PD1+ compared to PD1+ alone (**Figures 1B, C**).

We have previously shown that after islet inflammation is established, IGRP-specific CD8+ T cells expand in numbers and recirculate between islets and peripheral lymphoid organs (28). These T cells can be tracked using tetramers and phenotyped by flow cytometry. Almost all IGRP-specific CD8+ T cells in the islets expressed TIGIT (**Figures 1D, E**), but in the spleen, TIGIT was not expressed, while PD1 still remained high (**Figures 1D, F**). These results suggest that for sustained TIGIT expression, autoreactive T cells require continuous engagement with cognate antigen.

We next assessed TCF1+ PD1+ stem-like memory CD8+ T cells using the cell surface molecule SLAMF6 as a surrogate marker for TCF1 expression (11, 30). In the islets of NOD mice, the majority of PD1+ CD8+ T cells were SLAMF6+ and thus in a stem-like memory state (**Figure 1G**). Among PD1+ CD8+ T cells, more than 50% were SLAMF6+TIGIT+, while approximately 30% were SLAMF6+TIGIT− (**Figures 1G, H**). These results indicate that TIGIT is expressed on the majority of stem-like memory CD8+ T cells in the islets.

### CD226 and TIGIT are not co-expressed on islet-infiltrating CD8+ T cells

Although TIGIT exerts its inhibitory activity by competing with the co-stimulatory molecule CD226 for its ligand, it has been shown that CD226+TIGIT+ CD8+ T cells still retain their cytotoxic capacity because they have not differentiated into a completely dysfunctional state like CD226-TIGIT+ CD8+ T cells (31). Supporting this, CD226+TIGIT+ T cells in tumors respond better to immune checkpoint therapy (31–33). Hence, we examined the expression of CD226 together with TIGIT on islet-infiltrating T cells. Islet-specific stem-like memory T cells in NOD mice are functional because they can give rise to effector T cells, which kill beta cells; therefore, we expected the majority of CD8+ T cells to be CD226+TIGIT+. Surprisingly, we found that TIGIT and CD226 are co-expressed only in 7% of islet-infiltrating PD1+ CD8+ T cells (**Figures 1I, J**).

### scRNA sequencing identifies stem-like memory and terminally differentiated CD8+ T cells with TIGIT expression in the islets

**Figure 2:**
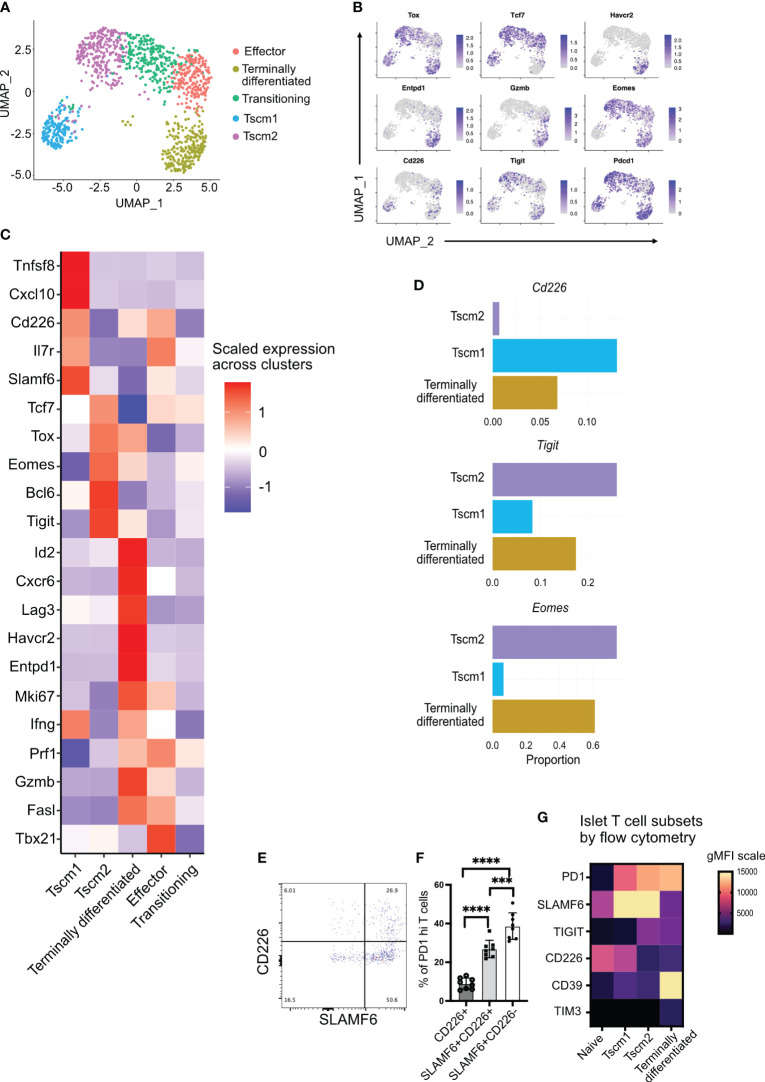
Tigit, Cd226, and Eomes expression in islet-infiltrating stem-like memory CD8+ T cells using single-cell RNA sequencing. **(A)** Uniform Manifold Approximation and Projection (UMAP) plot of islet-infiltrating PD1+CD44+ CD8+ T cells from 14–16-week-old NOD mice showing clusters of stem-like memory (Tscm1 and Tscm2), effector, terminally differentiated, and transitioning T cells. **(B)** Feature plot showing expression of indicated genes in various subsets of T cells. **(C)** Heatmap showing differentially expressed genes in Tscm1, Tscm2, terminally differentiated, and effector T cells. **(D)** The proportions of cells expressing the indicated genes in Tscm1, Tscm2, and terminally differentiated T cells. **(E, F)** Frequency of CD226 and SLAMF6 expressing PD1+CD8+ T cells from islets of 14–18-week-old female NOD mice. **(E)** Representative plot and **(F)** pooled data showing mean ± SD of individual mice (n=8), ***p<0.001 and ****p<0.0001, one-way ANOVA Tukey’s multiple comparison test. **(G)** Heatmap showing gMFI expression of cell surface markers using flow cytometry for the identification of T-cell subsets in the islets.

To investigate the heterogeneity and differentiation status of islet-infiltrating CD8+ T cells, we isolated T cells from the islets and performed scRNA sequencing. We focused on CD8+ T cells positive for *Pdcd1* (PD1) and *Cd44* expression to investigate activated, antigen-experienced T cells. These cells separated into five different clusters (**Figure 2A**). Two clusters were identified as stem-like memory T cells (Tscm1 and Tscm2) based on the expression of *Tcf7*, *Tox*, and *Pdcd1* (**Figures 2B, C**). Although both Tscm1 and Tscm2 expressed *Tox*, a master regulator of T-cell exhaustion (34–36) (**Figure 2B**), Tscm 2 had higher *Tox* expression compared to Tscm1 (**Figure 2C**). Tscm1 expressed higher levels of *Tnfsf8* (CD30L)*, Cxcl10*, and *Il7r*, whereas Tscm2 expressed higher levels of *Tcf7* and *Bcl6* (**Figure 2C**), key transcription factors associated with stem-memory or progenitor T cells (37).

Terminally differentiated T cells expressed multiple immune checkpoint molecules including *Tigit*, *Lag3*, *Entpd1* (CD39), and *Havcr2* (TIM3) (**Figures 2B, C**). These terminally differentiated T cells did not express *Tcf7* (TCF1) but expressed *Tox* (**Figures 2B, C**). They also expressed the transcription factor *Id2* (**Figure 2C**). Terminally differentiated cells and effector T cells expressed the cytotoxic molecules *Prf1* (perforin), *Gzmb* (granzyme B), and *Ifng* (**Figure 2C**). However, effector T cells expressed *Tcf7* and *Tbx21* (T-bet) but not *Tox* (**Figures 2B, C**). Effector T cells also expressed *Il7r* (**Figure 2C**), which marks effector T cells having the potential to develop as memory T cells (38). There was also a population of transitioning T cells between Tscm2 and effector cells (**Figure 2A**). These transitioning T cells expressed not only the effector molecule perforin (shared with the effector cluster) but also *Tcf7* (shared with effector and Tscm2 clusters) (**Figure 2C**). Our identification of stem-like memory T cells and terminally differentiated T cells in islets of NOD mice is consistent with results from other studies (7–9).

We next looked at the expression of *Cd226*, *Tigit*, and *Eomes*, a transcription factor that has been shown to downregulate *Cd226* expression in tumor-infiltrating T cells (31). Of the stem-like memory T-cell clusters, *Cd226* and *Eomes* expression was mutually exclusive in clusters Tscm1 and Tscm2, respectively (**Figures 2B, C**). There were few *Cd226*-expressing cells among all activated cells (**Figure 2B**). However, the *Cd226*-expressing cells were present in Tscm1 but not in Tscm2 (**Figures 2B–D**). In contrast, *Eomes* was expressed in all clusters except the *Cd226*-expressing Tscm1 (**Figure 2B**), and it was most highly expressed in Tscm2 (**Figures 2C, D**). The proportion of *Tigit*-expressing cells was highest in Tscm2 (**Figure 2D**). *Tigit* and *Eomes* were co-expressed in Tscm2 and terminally differentiated T cells (**Figures 2B, C**). A small number of *Cd226* and *Tigit* co-expressing cells was found in the Tscm1 cluster, while almost no *Cd226* and *Tigit* co-expressing cells were found in the Tscm2 cluster (**Figures 2B, C**). Flow-cytometry supported our scRNA data: the majority of SLAMF6+PD1+ stem-like memory T cells were TIGIT+ (corresponding to Tscm2) (**Figures 1G, H**), and very few of these cells were CD226+ (corresponding to Tscm1) (**Figures 2E, F**). Furthermore, we generated a heatmap of our flow cytometry data to confirm the scRNA annotation of T-cell subsets with protein expression. In the islets, PD1+CD44+ T cells were divided into Tscm1 (SLAMF6^hi^CD39^lo^CD226^hi^TIGIT^lo^), Tscm2 (SLAMF6^hi^CD39^lo^CD226^lo^TIGIT^hi^), and terminally differentiated (SLAMF6^lo^CD39^hi^CD226^lo^TIGIT^hi^) subsets (**Figure 2G**).

In summary, we identified two populations of stem-like memory T cells (Tscm1 and Tscm2) based on *Cd226*, *Tigit*, and *Eomes* expression. The Tscm2 cells were more similar to the terminally differentiated T cells with high *Tigit* and *Eomes* expression.

### CD226− EOMES+ islet-infiltrating CD8+ T cells retain their functionality

**Figure 3:**
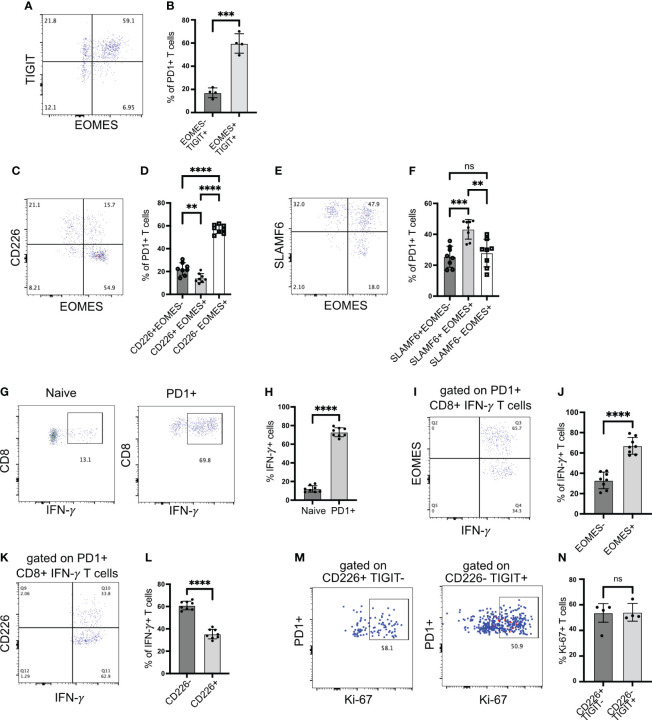
EOMES expression on islet-infiltrating T cells. **(A, B)** Frequency of EOMES+ and TIGIT+ cells among PD1+CD8+ T cells from islets. Representative plot **(A)** and pooled data showing mean ± SD and individual mice **(B)**. ***p<0.001, unpaired Student’s t-test. **(C, D)** Frequency of EOMES+ and CD226+ cells among PD1+CD8+ T cells from islets. Representative plot **(C)** and pooled data showing mean ± SD and individual mice **(D)**. One-way ANOVA Tukey’s multiple comparison test. **(E, F)** Frequency of SLAMF6 and EOMES-expressing cells among PD1+CD8+ T cells from islets, representative plot **(E)** and pooled data showing mean ± SD and individual mice **(F)**. One-way ANOVA Tukey’s multiple comparison test. **(G, H)** Frequency of IFN-γ producing CD8+ T cells from islets. Representative plots **(G)** and pooled data showing mean ± SD and individual mice **(H)**. Unpaired student’s t-test. **(I, J)** Frequency of EOMES-expressing IFN-γ producing cells among PD1+CD8+ T cells from islets. Representative plot **(I)** and pooled data showing mean ± SD and individual mice **(J)**. Unpaired Student’s t-test. **(K, L)** Frequency of CD226 expressing IFN-γ producing cells among PD1+CD8+ T cells from islets. Representative plots **(K)** and mean ± SD and individual mice **(L)**. Unpaired Student’s t-test. Female NOD mice 14–18 weeks old (n=8 mice/group) were used. p-values **p<0.01 and ****p<0.0001, ns=not significant. **(M, N)** Frequency of Ki-67+ cells among PD1+ cells gated on CD226+TIGIT− or CD226-TIGIT+ subsets. Representative plots **(M)** and mean ± SD and individual mice **(N)**. Unpaired Student’s t-test. Female NOD mice 14–18 weeks old (n=4 mice/group) were used.

The transcription factor *Eomes* is co-expressed with *Tigit*, but very few cells co-express *Eomes* with *Cd226* (**Figure 2B**). Hence, *Cd226*-negative *Eomes* expressing cells can be used to mark chronically stimulated *Tigit*-expressing T cells. We confirmed our scRNA-seq data using flow cytometry. Approximately 60% of PD1+CD8+ T cells from the islets co-expressed EOMES and TIGIT. Only 20% of cells were TIGIT+ EOMES− (**Figures 3A, B**). In addition, more the 80% of EOMES-expressing cells did not express CD226 (**Figures 3C, D**). Consistent with our scRNA-seq data, approximately 80% of EOMES+ cells were SLAMF6+ stem-like memory T cells (**Figures 3E, F**). Using EOMES staining, we were able to divide the SLAMF6+ stem-like memory cells into two populations, where SLAMF6+ EOMES+ cells resemble Tscm2 cells seen in the scRNA-seq data (**Figure 3E**).

The upregulation of EOMES and TIGIT in the absence of CD226 expression is linked with dysfunctional T cells and reduced proinflammatory cytokine production (31, 32). Hence, we investigated IFNγ production by islet-infiltrating T cells. When stimulated with PMA/ionomycin, more than 65% of PD1+CD8+ T cells produced IFNγ (**Figures 3G, H**). We expected that among IFNγ+ cells, the frequency of EOMES+ cells might be less because chronic antigen stimulation and EOMES expression are linked with the reduced capacity to produce cytokines. However, approximately 65% of IFNγ+ T cells were EOMES+, while approximately 30% of IFNγ+ T cells were EOMES− (**Figures 3I, J**). In addition, among IFNγ-producing CD8+PD1+ T cells, approximately 60% were CD226− (**Figures 3K, L**). We also examined proliferation of TIGIT+CD226− Tscm2 cells using Ki-67 as a marker as an additional readout of function. Both TIGIT-CD226+ and TIGIT+CD226− cell proliferated equally (**Figures 3M, N**). In summary, stem-like memory CD8+ T cells in autoimmune diabetes preserve their functional ability to produce cytokine and proliferate despite upregulating EOMES and losing CD226 expression.

### Beta cells and islet-infiltrating dendritic cells express the ligand for TIGIT, CD155

**Figure 4:**
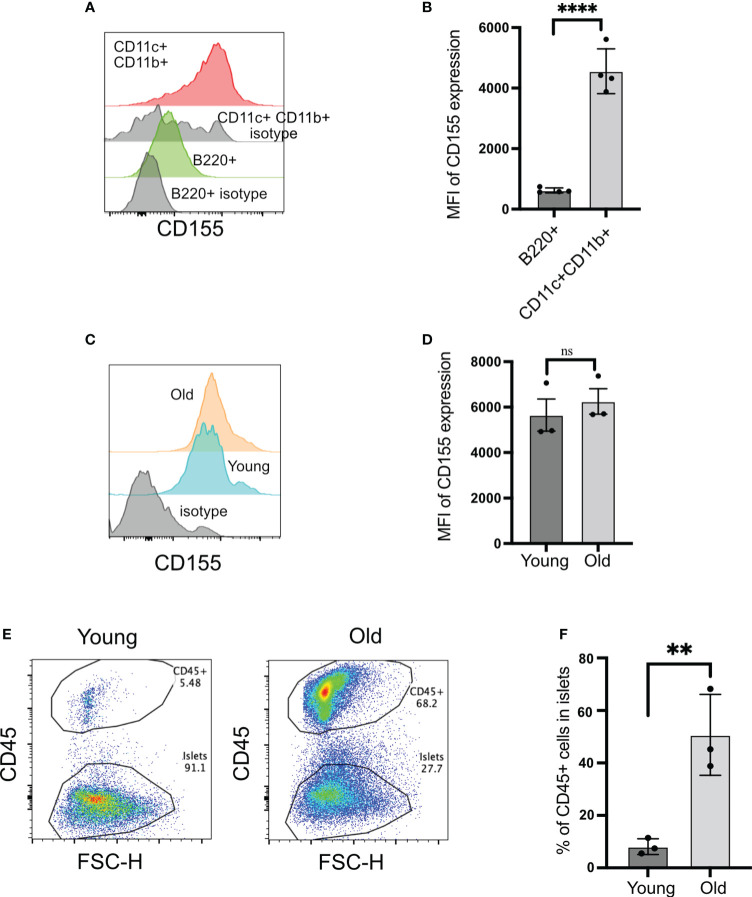
CD155 expression on beta cells and islet-infiltrating immune cells. **(A, B)** CD155 expression on B cells and CD11c+CD11b+ cells. Representative plots **(A)** and pooled data showing mean ± SD and individual mice of CD155 mean fluorescence intensity (MFI) (n=4 mice). Unpaired Student’s t-test. **(C, D)** CD155 expression on beta cells of young (4–6 weeks) or old (14–16 weeks) NOD mice. Representative plots **(C)** and pooled data showing mean ± SD and individual mice of CD155 MFI **(D)** (n=3 mice/group). Beta cells were identified as CD45− cells with high autofluorescence. Unpaired Student’s t-test. **(E, F)** Frequency of CD45+ cells in the islets of young and old NOD mice. Representative plots **(E)** and pooled data showing mean ± SD and individual mice (n=3 mice/group). Unpaired Student’s t-test. **p<0.01, ****p<0.0001, ns = not significant

TIGIT and CD226 engage with a common ligand, CD155, and inhibit or activate T cells, respectively. For TIGIT to act as an immune checkpoint in autoimmune diabetes, it needs to engage with CD155 in the islets. CD155 has been shown to be expressed by dendritic cells. In islets, CD155 was expressed at higher levels on the CD11b+CD11c+ dendritic cells compared to B cells (**Figures 4A, B**).

The expression of CD155 on beta cells is not known. CD155 expression on beta cells would provide a direct opportunity for TIGIT to engage with its ligand when T cells form an immunological synapse with beta cells. Beta cells expressed CD155 at high levels, and this was not dependent on the age of mice because both young and old mice exhibited similar levels of CD155 on their beta cells (**Figures 4C, D**). Only 8% of islet cells were CD45+ in the islets from young mice versus 50% of CD45+ cells in the old mice (**Figures 4E, F**). These findings suggested that CD155 expression does not depend on the status of islet inflammation, and there is the opportunity for TIGIT on CD8+ T cells to engage with its ligand both on dendritic cells and beta cells in the islets.

### TIGIT does not restrain stem-like memory T cells in autoimmune diabetes

T cells in NOD islets are restrained by immune checkpoints like PD1 (15) and LAG3 (19), which negatively regulate T-cell receptor signaling. In addition to competing with CD226 for its ligand CD155 to inhibit co-stimulation (23, 39), TIGIT can also negatively regulate T cells by inhibiting T-cell receptor signaling, independent of CD226 inhibition (24). Hence, we hypothesized that TIGIT might restrain TIGIT+CD226− stem-like memory T cells in autoimmune diabetes by negatively regulating T-cell receptor signaling.

**Figure 5:**
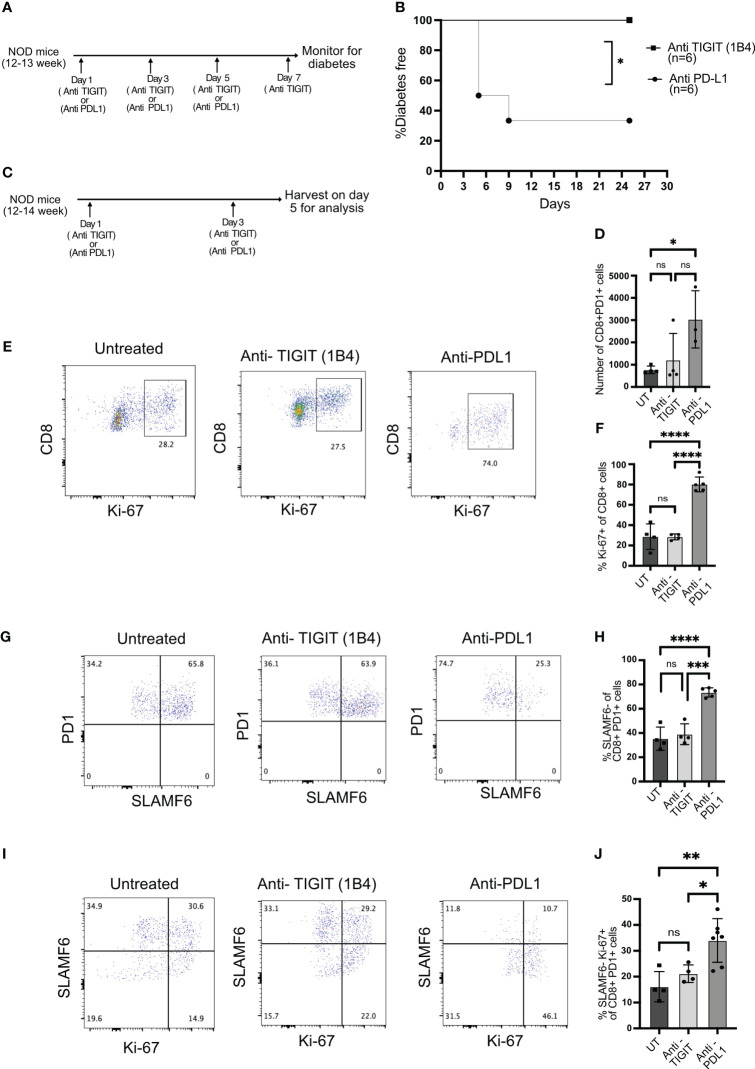
The role of TIGIT as an immune checkpoint in autoimmune diabetes. **(A)** Schematic of the treatment strategy for anti-TIGIT and anti-PDL1 antibodies. **(B)** Diabetes induction by anti-TIGIT antibody given on days 1, 3, 5, and 7, or anti-PDL1 antibody given on days 1, 3, and 5 to 12–13-week-old female NOD mice (n=6 mice/group). Statistical analysis was done by log rank (Mantel–Cox test). **(C)** Schematic of the treatment strategy to evaluate the impact of anti-TIGIT and anti-PDL1 on islet-infiltrating T cells. **(D)** The total number of CD8+PD1+ T cells and **(E)** the frequency of Ki-67 expressing cells among CD8+ T cells from the islets of untreated (UT), anti-TIGIT, and anti-PDL1 treated 12–14-week-old female NOD mice. Representative plots **(E)** and pooled data showing mean ± SD and individual mice **(F)** (n=4–5 mice/group). One-way ANOVA Tukey’s multiple comparison test. **(G, H)** The frequency of PD1+ and SLAMF6+ among PD1+ CD8+ T cells from islets after anti-TIGIT or anti-PDL1 treatment of NOD mice. Representative plots **(G)** and pooled data showing mean ± SD and individual mice **(H)** (4–5 mice/group). One-way ANOVA Tukey’s multiple comparison test. **(I, J)** The frequency of SLAMF6-Ki67+ cells among PD1+CD8+ T cells from the islets after anti-TIGIT or anti-PDL1 treatment. Representative plots **(I)** and pooled data showing mean ± SD and individual mice **(J)** (n=4-7 mice/group). p-values *p<0.05,**p<0.01,***p<0.001, and ****p<0.0001, ns=not significant.

To test this, we inhibited TIGIT using a blocking antibody (1B4) (29) in 12–14-week-old NOD mice when TIGIT+ T cells are present in the islets. As a comparison, we blocked PD1 signaling with anti-PDL1 antibody and investigated its impact on diabetes induction. TIGIT blockade did not induce autoimmune diabetes, while PDL1 inhibition resulted in rapid diabetes onset in NOD mice (**Figures 5A, B**).

Blocking immune checkpoint molecules results in the proliferation and differentiation of stem-like memory T cells into terminally differentiated effector like T cells (8, 13). After two doses of anti-PDL1 or anti-TIGIT antibody treatment, islets were isolated, and T cells were analyzed for their proliferation and differentiation status (**Figure 5C**). If the immune checkpoint is acting on stem-like memory T cells, its inhibition should result in proliferating terminally differentiated T cells derived from stem-like memory T cells. Anti-PDL1 treatment resulted in the rapid proliferation of CD8+ T cells and increase in the number of CD8+PD1+ T cells, but the majority of cells from anti-TIGIT treated mice remained Ki-67 negative, similar to untreated mice (**Figures 5D–F**). PDL1 but not TIGIT blockade induced the conversion of stem-like memory T cells into terminally differentiated cells as marked by an increase in the frequency of SLAMF6− PD1+ T cells (**Figures 5G, H**). Consistent with the above findings, PD1 inhibition increased the frequency of SLAMF6− Ki-67+ T cells, but TIGIT inhibition was not different to untreated mice, indicating no differentiation of stem-like memory T cells after TIGIT inhibition (**Figures 5I, J**).

### TIGIT acts as an immune checkpoint in autoimmune diabetes in the absence of PD1 signaling

Recent studies of tumor-infiltrating T cells showed that the success of PD1 or TIGIT inhibition as immunotherapy depends on the presence of CD226 and TIGIT co-expressing T cells in the tumor (33).

In the islets of NOD mice, we did not find many CD226+TIGIT+ T cells, and this could be a reason why TIGIT was not acting as an immune checkpoint in NOD mice.

**Figure 6:**
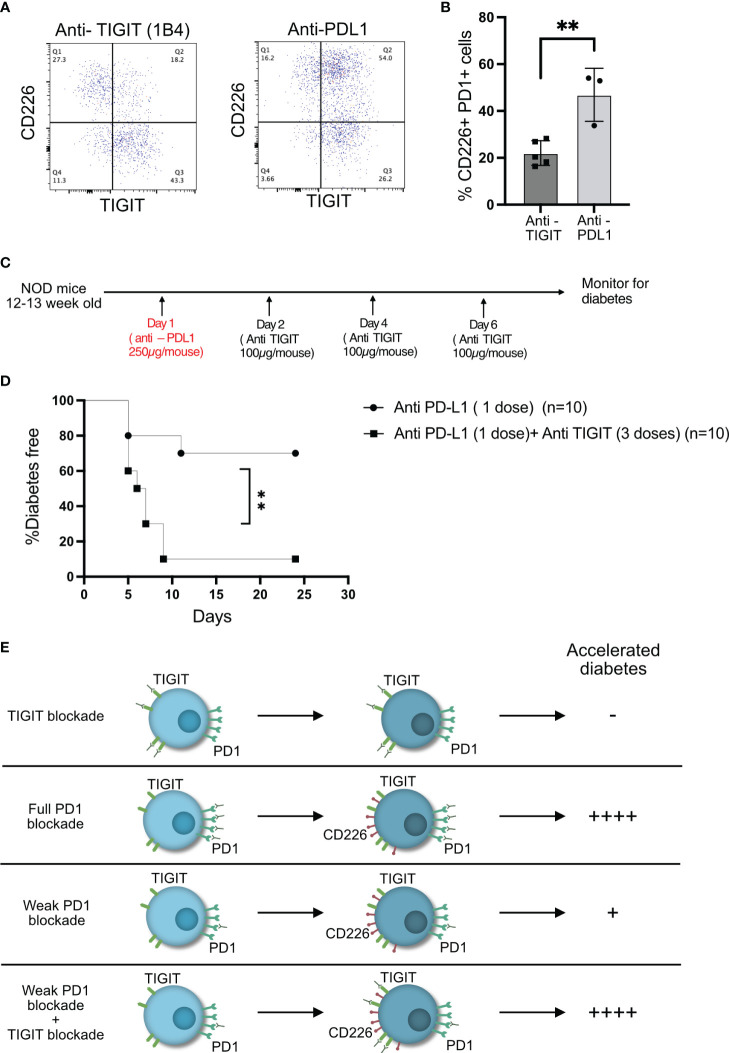
TIGIT acts as an immune checkpoint in the absence of PD1 signaling. **(A, B)** Frequency of CD226 and TIGIT-expressing PD1+CD8+ T cells from the islets of NOD mice after two doses of anti-PDL1 treatment. Representative plots **(A)** and pooled data showing mean ± SD and individual mice (n=3–5 mice/group). Unpaired Student’s t-test. **(C)** Schematic of the treatment strategy for combining anti-TIGIT and anti-PDL1 antibodies. **(D)** Diabetes incidence after one dose of anti-PDL1 alone or in combination with anti-TIGIT as detailed in the schematic in **(C)**. Statistical analysis was done by log rank (Mantel–Cox test). p-values **p<0.01. **(E)** Schematic of effect on diabetes as a result of full or weak PD1 blockade or weak PD1 blockade together with TIGIT blockade.

We hypothesized that anti-PDL1 therapy might result in increased frequency of CD226+TIGIT+ T cells in the islets, and this would result in the right conditions for TIGIT to act as an immune checkpoint in the islet-infiltrating T cells. Two doses of anti-PDL1 led to a significant rise in CD226+TIGIT+ T cells (**Figures 6A, B**). A single dose of anti-PDL1 in 12–13-week-old NOD mice induced rapid diabetes in approximately 30% of the mice, while one dose of anti-PDL1 followed by three doses of TIGIT blocking antibody resulted in rapid diabetes in 90% of NOD mice (**Figures 6C, D**). This result suggests that TIGIT acts as a secondary immune checkpoint to restrain beta-cell-specific autoreactive T cells but only in the absence of PD1 signaling (**Figure 6E**).

## Discussion

Despite the progress in developing novel immune therapies for cancer, it is poorly understood how various immune checkpoints operate in autoimmune diseases. Here, we used the NOD mouse model to study the role of the immune checkpoint TIGIT in autoimmune diabetes. Our data show that despite being expressed on autoreactive T cells and its ligand being present in islets, TIGIT does not act as a primary immune checkpoint in autoimmune diabetes. TIGIT was upregulated on islet-infiltrating T cells, and using scRNA seq, we identified a stem-like memory T-cells subset in the islets based on TIGIT expression. However, TIGIT restrained these stem-like memory T cells in the islets when PD1 signaling was reduced, resulting in rapid diabetes when TIGIT is blocked. Our results complement emerging data, suggesting a context-dependent functional hierarchy, synergy, or redundancy between immune checkpoints.

Our scRNA-seq analysis identified major stem-like memory, terminally differentiated, and activated T-cell subsets in agreement with the previously published scRNA data from NOD islets (7–9). These subsets were also identified in IGRP tetramer+ T cells from pancreatic lymph nodes of NOD mice (40). We also showed that the Tscm (also called T_PEX_) and terminally differentiated (also called T_EX_) cells from NOD mice have enriched gene signatures of classic Tscm and T_EX_ cells found in other models (9). Unlike previous studies, our manual annotation of the data identified two stem-like memory subsets, Tscm1 and Tscm2, based on *Eomes*, *Cd226*, and *Tigit* expression. These were likely missed in the previously published studies due to the challenges of annotation of scRNA-seq data and the relatively low expression of *Cd226*.

TIGIT is expressed at high levels on terminal differentiated T cells in chronic LCMV (41) infection and tumors (11), and the PD1+TIGIT+ phenotype is normally linked with hyporesponsive CD8+ T cells. TIGIT is also expressed on transitory effector T cells and TCF1+ stem-like memory T cells in chronic LCMV infection (42). In addition, during chronic LCMV infection, TIGIT+ T cells produce immunoregulatory cytokine IL-10 and limit immune pathology (43). Interestingly, in autoimmune diabetes, TIGIT-expressing stem-like memory T cells were found in the islets. Chronic antigen stimulation and EOMES-induced loss of CD226 have been linked with dysfunctional T cells. However, in the islets, T cells with a similar phenotype retain their functionality. This could be due to their unique stem-like memory differentiation state resulting from a TCF1-driven epigenetic program. PD1+ TIGIT+ stem-like memory T cells have also been identified as a human CD8+ T-cell subset that has been shown to be less functional (44). Interestingly, Teplizumab, which engages CD3 molecules on T cells, has been shown to increase the frequency of PD1+TIGIT+ T cells, and it was positively co-related with responders in the type 1 diabetes prevention trial (45). However, it remains to be determined if PD1+ TIGIT+ uniquely differentiated stem-like memory T cells exist in human type 1 diabetic islets and what is their functional status.

We found that unlike PD1, TIGIT is not acting as a primary immune checkpoint in stem-like memory T cells. CD226 knockout NOD mice had a reduced incidence of autoimmune diabetes (25). It was suggested that in the absence of co-stimulation via CD226, T-cell activation and effector/memory response were impaired leading to protection from autoimmune diabetes. In the same study, TIGIT knockout NOD mice developed autoimmune diabetes with the same incidence as wild-type NOD mice (25). It is possible that knocking out TIGIT from birth might be compensated by other immune checkpoints. However, our experiments using a TIGIT-blocking antibody after T cells infiltrate the islets confirm that inhibiting TIGIT alone cannot unleash self-reactive T cells in the islets.

One of the ways that TIGIT imparts its inhibitory activity is by competing with CD226 for the ligand CD155. For this activity, both CD226 and TIGIT need to be co-expressed on the same T cells. Our data show that the majority of the islet-infiltrating CD8+ T cells did not co-express CD226 and TIGIT. However, blocking PD1 signaling increased CD226 and TIGIT co-expressing cells, and TIGIT inhibition led to rapid diabetes in NOD mice. This finding establishes a hierarchy of immune checkpoints in autoimmune diabetes where TIGIT is downstream of PD1 in negatively regulating beta-cell-specific T cells. In line with a recent study of tumor-infiltrating T cells (33), these results also suggest that inhibiting CD226 signaling by TIGIT might be one of the major mechanisms by which TIGIT imparts its immune regulation in self-reactive T cells. This can be addressed by future studies in autoimmune diabetes.

We showed that CD155, a ligand for TIGIT and CD226, is expressed on the beta cells and islet-infiltrating dendritic cells. This is in agreement with previous data showing CD155 expression on human stem-cell-derived beta cells (46). Despite this, it remains possible that TIGIT is not engaged with its cognate ligand in the islets. Indeed, a limitation of our study is concrete evidence of TIGIT’s engagement with CD155, either on beta cells or dendritic cells. As yet, no distinctive gene signature or markers of TIGIT engagement on T cells has been identified. Future studies on the intracellular signaling pathways of TIGIT will identify markers to enable us to confirm TIGIT engagement in NOD mice. TIGIT engagement with CD155 imparts immunoregulatory signaling in dendritic cells (20), and in melanoma, TIGIT-CD155 interaction has been shown to reduce cytotoxic T-cell responses (47). It is not known if dendritic cells can also protect themselves from cytotoxic T cells using similar mechanisms or whether this has any impact on antigen presentation in cancer or autoimmunity.

Our study has some limitations. Although our data suggested that TIGIT can act as an immune checkpoint when PD1 is inhibited, we cannot conclude that TIGIT is acting as an immune checkpoint on the same cell where PD1 is inhibited. Similarly, it is not possible to infer if TIGIT is acting as an immune checkpoint on stem-like memory T cells or if it exerts its immune checkpoint activity on another T-cell subset that has been induced because of PD1 inhibition. From our results, it is difficult to conclude whether TIGIT acts as an immune checkpoint via exclusively negatively regulating CD226. This could be addressed further using CD226 and TIGIT knockout mice. TIGIT is also expressed on islet-infiltrating CD4+ T cells, and from our data, we cannot rule out the impact of TIGIT on CD4+ T cells.

In summary, TIGIT-expressing stem-like memory T cells are present in autoimmune diabetes, but TIGIT acts as a secondary immune checkpoint in restraining beta-cell-specific T cells. This study established an order of immune checkpoint molecules in autoimmune diabetes by showing that TIGIT can act as an immune checkpoint only in the absence of PD1. We have identified subsets of stem-like memory T cells based on the expression of TIGIT, CD226, and EOMES. Our data suggest that engaging TIGIT might not be a suitable way to target these cells in autoimmune diabetes because it is downstream of PD-1, which might be a better target for successful immunotherapy. It is possible that other immune checkpoint molecules also targeting molecules that are upstream in the hierarchy of immune checkpoints might provide a better chance for successful immunotherapy for autoimmune diabetes. Fundamental knowledge of how immune checkpoint receptors function in autoimmunity will be helpful in developing future immunotherapies where boosting immune checkpoint signaling could prevent autoimmune diabetes.

## Data availability statement

The datasets presented in this study can be found in online repositories. The names of the repository/repositories and accession number(s) can be found below: https://www.ncbi.nlm.nih.gov/geo/, GSE247956.

## Ethics statement

The animal study was approved by St Vincent’s Hospital Animal Ethics Committee. The study was conducted in accordance with the local legislation and institutional requirements.

## Author contributions

PT: Conceptualization, Data curation, Formal Analysis, Investigation, Methodology, Validation, Writing – original draft, Writing – review & editing, Project administration, Visualization. GJ: Data curation, Formal Analysis, Investigation, Writing – review & editing. DD: Data curation, Formal Analysis, Investigation, Writing – review & editing. CC: Data curation, Formal Analysis, Investigation, Visualization, Writing – review & editing. CS: Formal Analysis, Investigation, Writing – review & editing. TG: Formal Analysis, Investigation, Writing – review & editing. TC: Formal Analysis, Investigation, Writing – review & editing. LE: Formal Analysis, Investigation, Writing – review & editing. LB: Resources, Writing – review & editing. NJ: Resources, Writing – review & editing. TK: Conceptualization, Funding acquisition, Supervision, Writing – review & editing. HT: Conceptualization, Funding acquisition, Project administration, Resources, Supervision, Validation, Visualization, Writing – original draft, Writing – review & editing. BK: Conceptualization, Project administration, Supervision, Writing – review & editing.
