# Supplementary material for: TIGIT acts as an immune checkpoint upon inhibition of PD1 signaling in autoimmune diabetes
Source: Front Immunol. 2024 Mar 12;15:1370907. doi: 10.3389/fimmu.2024.1370907 (PMC10964479; doi:10.3389/fimmu.2024.1370907)
Supplement: Supplementary file 1 [file DataSheet_1.docx]

***Supplementary Material***

**TIGIT acts as an immune checkpoint upon inhibition of PD1 signaling in autoimmune diabetes**

Prerak Trivedi ^1^, Gaurang Jhala ^1^, David J De George ^1,2^, Chris Chiu ^1^, Claudia Selck ^1,2^, Tingting Ge ^1,2^, Tara Catterall ^1^, Lorraine Elkerbout ^1^, Louis Boon ^3^, Nicole Joller ^4^, Thomas W Kay ^1,2^, Helen E Thomas ^1,2*^, Balasubramanian Krishnamurthy ^1,2^

**Supplementary Figures**

**
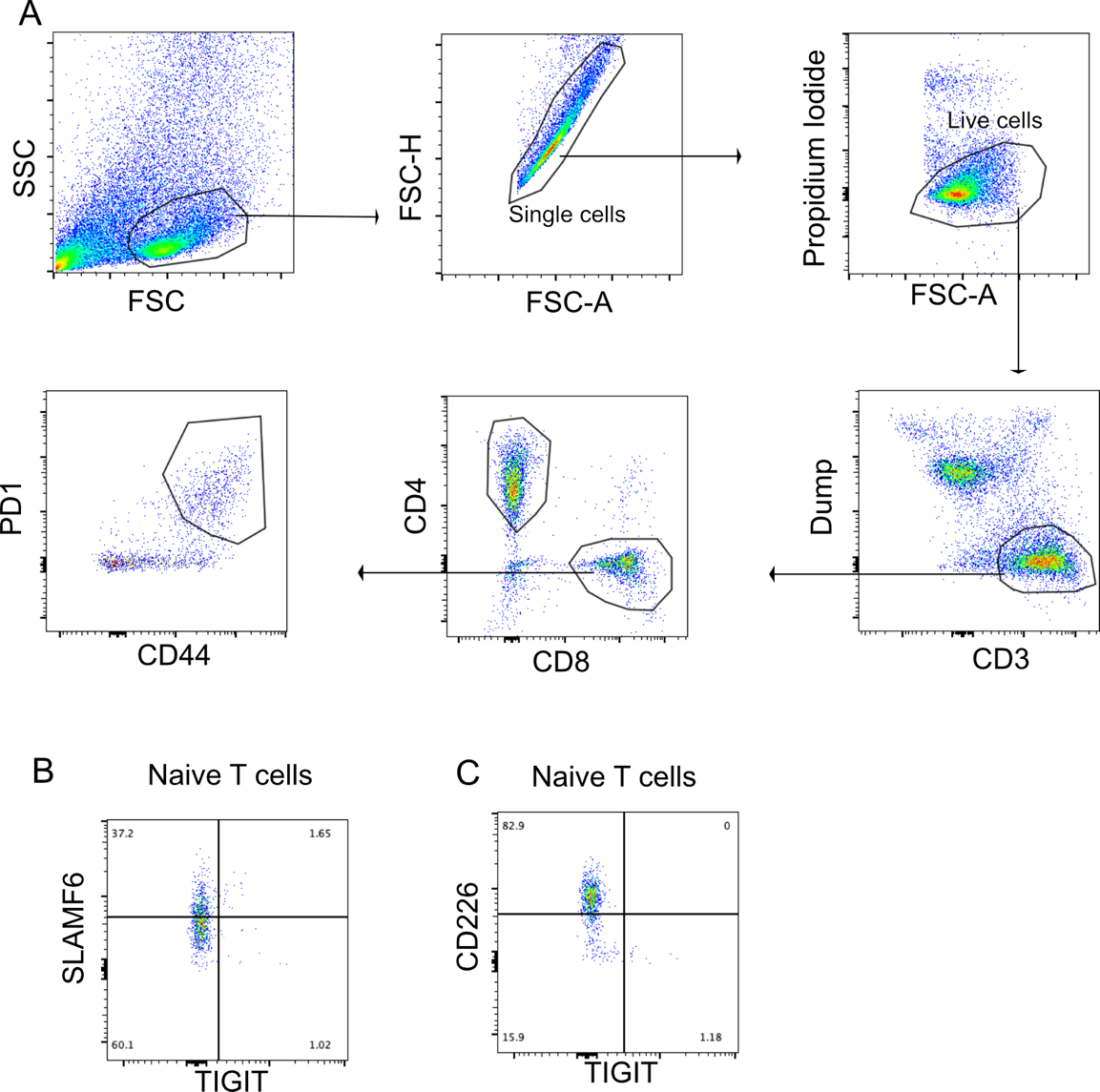
**

**Supplementary Figure 1. Gating strategy and gating controls for islet infiltrating CD8+ T cells.** (A) Gating strategy for analyzing (PD1+CD44+) CD8+ T cells from islets. (B) SLAMF6 and TIGIT and (C) CD226 and TIGIT expression on Naïve (PD1-CD44-) CD8+ T cells from islets.


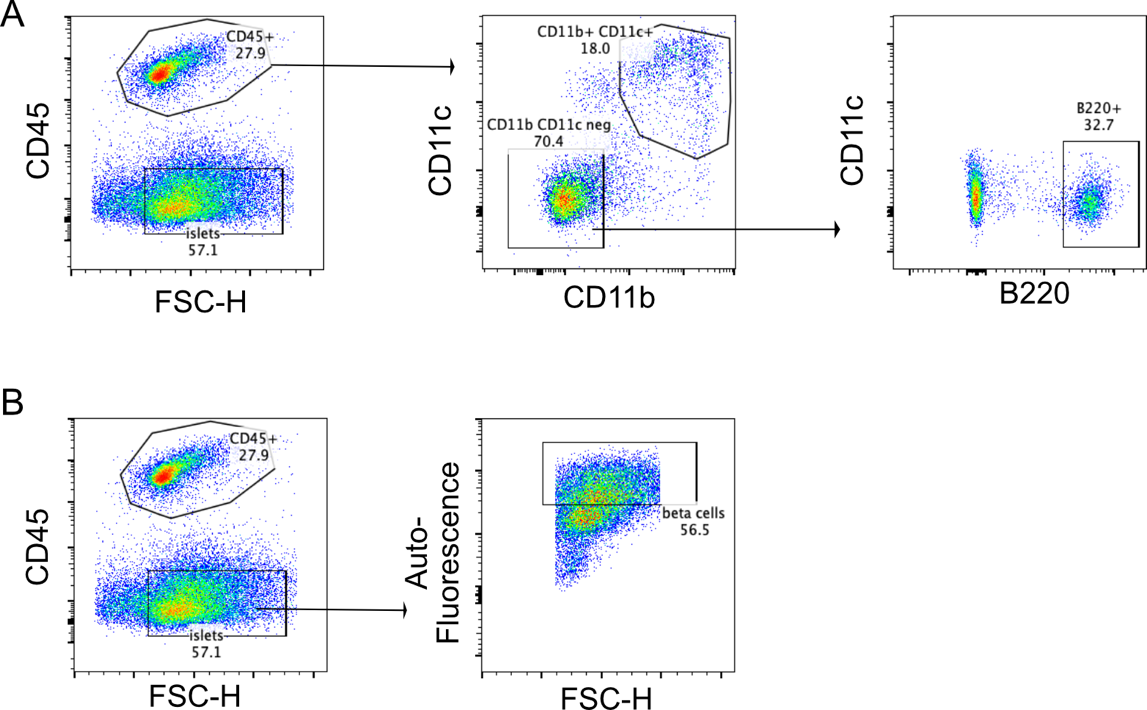


**Supplementary Figure 2. Gating strategy for the identification of B cells, dendritic cells and beta cells.** (A) CD45+ cells from islets are further divided into dendritic cells (CD11b+CD11c+) or B cells (B220+). (B) Beta cells gated as CD45- cells with high autofluorescence.

**Supplementary Table 1**

Antibodies used in this study

| Target | Fluorophore | Clone | Manufacturer |
| --- | --- | --- | --- |
| Cd11c | eF450 | M1/70 | Invitrogen |
| Cd11b | eF450 | N41B | Invitrogen |
| Ly6G | eF450 | RB6-8C5 | Invitrogen |
| B220 | eF450 | RA3-6B2 | Invitrogen |
| CD3 | V500 | 500A2 | BD Biosciences |
| CD4 | APC-Cy7 | GK1.5 | BD Pharmigen |
| CD8 | APC | 53-6.7 | BioLegend |
| CD8 | BV711 | 53-6.7 | BioLegend |
| CD8 | APC-Cy7 | 53-6.7 | BD Pharmingen |
| PD-1 | BV605 | 29F.1A12 | BioLegend |
| CD44 | PE-Cy7 | IM7 | BioLegend |
| TIM3 | AF700 | 215008 | R&D Systems |
| SLAMF6 | FITC | REA1097 | Miltenyi Biotech |
| CD226 | BV785 | TX42.1 | BioLegend |
| TIGIT | PE/dazzle | 1G9 | BioLegend |
| TIGIT | eF660 | GIGD7 | eBioscience |
| CD39 | BV711 | Y23-1185 | BioLegend |
| EOMES | PE-Cy7 | Dan11mag | eBioscience |
| EOMES | PE-eF610 | Dan11mag | eBioscience |
| Ki-67 | PE-Cy7 | SolA15 | eBioscience |
| IFN-γ | FITC | XMG1.2 | eBioscience |
| CD155 | BV605 | TX56 | BioLegend |
| CD11c | APC | HL3 | BD Pharmigen |
| B220 | FITC | RA3-6B2 | BD Pharmigen |
| CD45 | PE-Cy7 | 30-F11 | BD Pharmigen |
